# Host Immune Transcriptional Profiles Reflect the Variability in Clinical Disease Manifestations in Patients with *Staphylococcus aureus* Infections

**DOI:** 10.1371/journal.pone.0034390

**Published:** 2012-04-04

**Authors:** Romain Banchereau, Alejandro Jordan-Villegas, Monica Ardura, Asuncion Mejias, Nicole Baldwin, Hui Xu, Elizabeth Saye, Jose Rossello-Urgell, Phuong Nguyen, Derek Blankenship, Clarence B. Creech, Virginia Pascual, Jacques Banchereau, Damien Chaussabel, Octavio Ramilo

**Affiliations:** 1 UT Southwestern Medical Center, Dallas, Texas, United States of America; 2 Baylor Institute for Immunology Research, Dallas, Texas, United States of America; 3 Nationwide Children's Hospital, Columbus, Ohio, United States of America; 4 Vanderbilt University School of Medicine, Nashville, Tennessee, United States of America; 5 Hoffmann-Laroche, Nutley, New Jersey, United States of America; 6 Benaroya Research Institute, Seattle, Washington, United States of America; National Institutes of Health, United States of America

## Abstract

*Staphylococcus aureus* infections are associated with diverse clinical manifestations leading to significant morbidity and mortality. To define the role of the host response in the clinical manifestations of the disease, we characterized whole blood transcriptional profiles of children hospitalized with community-acquired *S. aureus* infection and phenotyped the bacterial strains isolated. The overall transcriptional response to *S. aureus* infection was characterized by over-expression of innate immunity and hematopoiesis related genes and under-expression of genes related to adaptive immunity. We assessed individual profiles using modular fingerprints combined with the molecular distance to health (MDTH), a numerical score of transcriptional perturbation as compared to healthy controls. We observed significant heterogeneity in the host signatures and MDTH, as they were influenced by the type of clinical presentation, the extent of bacterial dissemination, and time of blood sampling in the course of the infection, but not by the bacterial isolate. System analysis approaches provide a new understanding of disease pathogenesis and the relation/interaction between host response and clinical disease manifestations.

## Introduction

*Staphylococcus aureus* has emerged as one of the most frequent cause of community-acquired invasive bacterial infections, with significant morbidity and mortality. Just in 2005 in the United States, 18,650 deaths were reported due to methicillin-resistant *S. aureus* (MRSA) [1], [2], [3], [4], [5], [6]. The spectrum of community-associated (CA) *S. aureus* disease is wide and patients can present with a variety of clinical illness, ranging from mild soft tissue infections to invasive disease such as bacteremia, pneumonia, or osteoarticular infections [1], [7], [8]. The emergence of multidrug resistant *S. aureus* strains worldwide [9], [10] combined with limited therapeutic options demand novel approaches to further elucidate host-pathogen interactions, and especially host responses to *S. aureus* infection. Circulating leukocytes represent an accessible source of molecular information, which can be studied by analyzing the whole blood genome-wide transcriptome. The value of blood microarray analysis has been illustrated in infection, cancer and autoimmunity [11], [12], [13], [14], leading to diagnostic and therapeutic advances [14].

We have previously used microarray analysis to characterize the differences in host responses to different microbial pathogens [11] including *S. aureus* (Ardura et al, 2009) by analyzing peripheral blood mononuclear cells (PBMC) from pediatric patients hospitalized with acute infections. The neutrophil depletion from PBMC may result in the loss of key information in the systemic characterization of bacterial infections [15]. As new research tools became available we performed whole blood microarray profiling –including neutrophils – in a new cohort of 99 pediatric patients with *S aureus* infection. Using this large cohort we now defined: a) the common host response to *S aureus* infection; and b) the differences in host response patterns depending on the site of infection and the clinical presentation of the disease. The main objective of this analysis was to determine whether the transcriptional profiles reflect the variation of clinical disease manifestations and to provide new insights in disease pathogenesis by defining the differences in host responses among patients with different clinical presentations.

## Results

### *S. aureus* induces a distinct and robust transcriptional signature in whole blood

**Figure 1:**
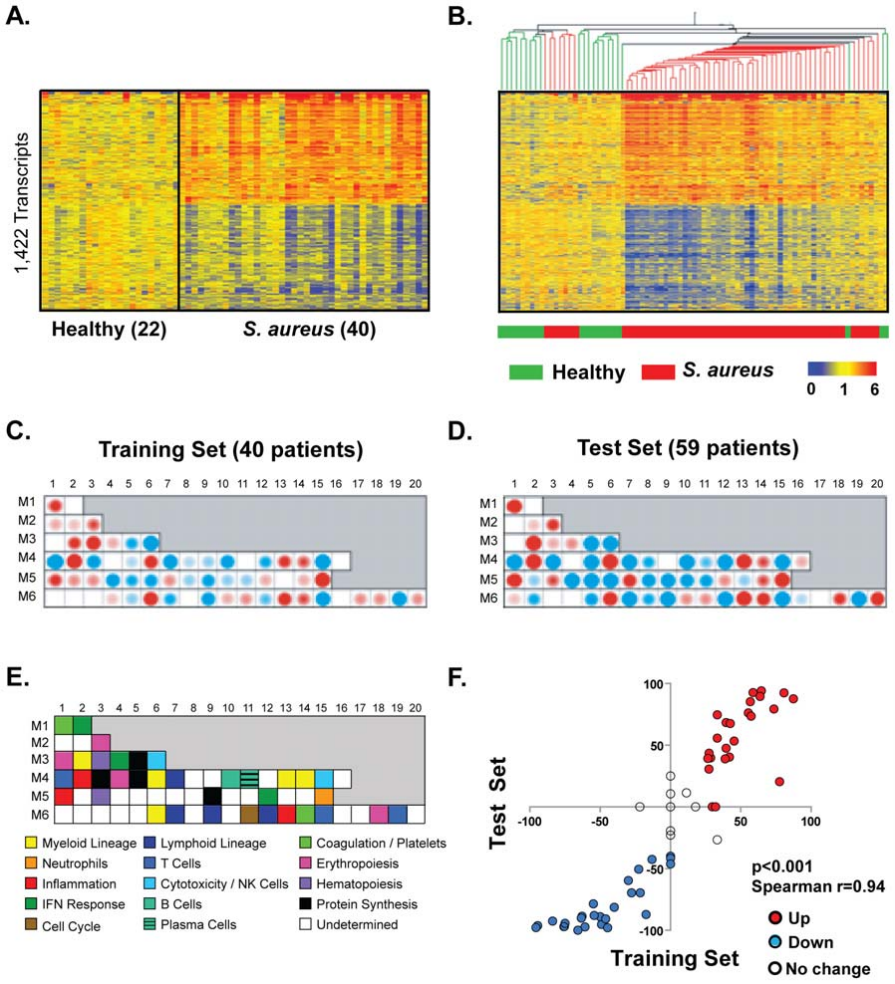
The *S. aureus* infection whole blood transcriptional signature is characterized by over-expression of myeloid lineage transcripts and under-expression of lymphoid lineage transcripts. **A.** Statistical group comparison between 22 healthy subjects and 40 patients with acute *S. aureus* infection (non-parametric test, α = 0.01, Benjamini-Hochberg multiple testing correction, 1.25 fold change) yielded 1,422 differentially expressed transcripts. Transcripts were organized by hierarchical clustering (Spearman) according to similarities in expression profiles. Each row represents a transcript and each column an individual subject. Normalized log ratio levels are indicated by red (over-expressed) or blue (under-expressed), as compared to the median of healthy controls. **B.** The same 1,422 transcript list and hierarchical clustering were applied to an independent test set of 22 healthy controls and 59 patients with acute *S. aureus* infection. Sample hierarchical clustering (Spearman) was performed on the 1,422 transcript list in the test set. **C.** Average modular transcriptional fingerprrint for *S. aureus* patients as compared to healthy controls in the training set. **D.** Average modular transcriptional profile for *S. aureus* patients as compared to healthy controls in the test set. **E.** Module functional annotations legend. **F.** Scatter plot comparing module expression between training (x-axis) and test (y-axis) sets. Spearman correlation was applied.

To define the whole blood biosignature of *S. aureus* infection in children, 99 patients and 44 healthy controls were assigned to two independent groups of subjects, to serve as “training” and “test” sets. The training set was used to identify the signature of *S. aureus* infection. This signature was validated in the independent test set by assessing its capacity to separate the patients from healthy controls by hierarchical clustering. The training set included 40 patients with *S. aureus* infection and 22 healthy controls, matched for age, sex and race (Table 1). Statistical group comparison yielded 1,422 differentially regulated transcripts. Hierarchical clustering of these transcripts grouped them according to similarities in gene expression patterns (Figure 1A). This signature was validated in the independent test set of 59 patients and 22 healthy controls (Figure 1B). Hierarchical clustering of these 1,422 transcripts grouped 52 out of 59 patients from the test set together. The seven patients who clustered with controls presented with either mild or moderate disease, and were closer to recovery and hospital discharge compared with all other patients.

**Table 1:** Demographic and laboratory characteristics of patients and healthy controls in training and test sets.

*Parameter*	TRAINING SET			TEST SET		
	*Patients*	*Controls*	*p^a^*	*Patients*	*Controls*	*p^a^*
Count	40	22		59	22	
Age (years)	8.00 [0.15–17]	7.0 [4]–[18]	0.865	6.50 [0.06–16]	3 [0.17–14.00]	0.03
Race	19AA,12H,9C	6AA,11H,4C,1O	0.156	19AA,18H,15C,7O	1AA,13H,6C,2O	0.018
Gender	20M,20F	12M,10F	0.795	34M,25F	11M,11F	0.805
WBC (10^3^/mm^3^)	9.3 [4.1–28.1]	6.8[3.40–12.6]	0.006	10.10[3.20–33.3]	9.1 [4.20–16.0]	0.065
Neutrophils (%)	52.0 [15–87]	50.7 [34.7–59.8]	0.536	55.00[12.0–84]	35.0 [10.0–55.00]	<0.001
Neutrophils (10^3^/mm^3^)	5.20 [1.16–22.4]	3.58[1.46–6.94]	0.025	5.63[0.74–18.1]	3.0 [0.84–7.76]	<0.001
Lymphocytes (%)	28.50 [5–67]	37.0 [29.4–53.8]	0.073	33.00 [5.00–72.0]	55.0[33.2–81.0]	<0.001
Lymphocytes (10^3^/mm^3^)	2.70[0.93–11.0]	2.72 [1.29–4.36]	0.721	3.42 [0.44–13.3]	5.3 [1.44–11.2]	0.016
Monocytes (%)	7.50[1]–[21]	7.30[5.00–10.6]	0.775	8.0 [2]–[17]	5.6 [2.00–9.0]	0.096
Monocytes (10^3^/mm^3^)	0.83 [0.08–3.40]	0.48 [0.19–0.72]	0.036	0.75 [0.14–3.49]	0.44 [0.30–0.98]	0.01
RBC (10^6^/mm3)	3.92 [2.6–4.7]	3.90[3.40–4.03]	0.507	4.05 [1.27–5.65]	no data	
Hemoglobin	10.5 [7.3–13.7]	8.80[7.90–11.40]	0.1	10.60 [3.4–13.7]	12.60*	
Hematocrit (%)	31.70 [22.1–38.6]	27.25[25.90–33.30]	0.13	31.70[11.1–40.3]	36.6*	
MCV (um^3^)	80.90 [70.7–88.9]	73.50[67.00–86.00]	0.083	81.00 [56.8–97.6]	no data	
MCH (pg)	27.30 [22.9–29.8]	23.75[20.40–29.30]	0.03	27.00 [17.9–33.4]	no data	
MCHC (g/dL)	33.6 [31–35.7]	32.05[30.40–34.20]	0.015	33.5 [30.6–35.9]	no data	
RDW (%)	13.40[11.8–19.4]	12.75[11.50–14.90]	0.337	13.30 [11.6–17.6]	no data	
MPV	9.30 [7.7–15]	7.80[6.50–10.00]	0.009	9.50 [7.8–12.4]	no data	
Platelets (10^3^/mm^3^)	412 [83–880]	261.00[214.00–288.00]	0.005	414.00 [136–833]	335.00*	
ESR	67.00 [15–142]	no data		68.00 [8–135]	no data	
CRP (mg/dL)	5.95 [0.1–37.4]	no data		4.30 [0.1–42.8]	no data	
Hospitalization Duration	11.00 [5–98]	n/a		9.00 [1–53.00]	n/a	
Draw Day	5.00 [2]–[16]	n/a		5.00 [1–35]	n/a	
Draw Index	0.43 [0.04–1]	n/a		0.50 [0.08–1]	n/a	

Median values [min-max range]; AA = African-American, C = Caucasian, H = Hispanic, O = Other; M = Male, F = Female; WBC = White Blood Count; RBC = Red Blood Cell count; MCV = Mean Corpuscular Volume; MCH = Mean Corpuscular Hemoglobin; MCHC = Mean Corpuscular Hemoglobin Concentration; RDW = Red blood cell Distribution Width; MPV = Mean Platelet Volume; ESR = Erythrocyte Sedimentation Rate; CRP = C-reactive Protein;

* only 1 sample.

Statistics: for categorical variables, Fisher's exact test is used; for continuous variables, Mann-Whitney test is used.

### *S. aureus* infection activates innate immunity and suppresses the adaptive immune response

To better understand the whole blood response to *S. aureus* infection and the immune pathways activated, we used an analytical framework of 62 transcriptional modules that group together genes with shared expression pattern and similar biological function across independent blood transcriptional datasets [16]. Module maps were derived independently for the training (Figure 1C) and test sets (Figure 1D), using their respective healthy control group as reference. Patients with acute *S. aureus* infection demonstrated significant over-expression of modules linked to the myeloid lineage (M3.2, M4.6, M4.13, M4.14, M6.6), and inflammation (M4.2, M5.1, M6.13) confirming our earlier findings in PBMC. Furthermore, patients displayed over-expression of modules linked to the coagulation cascade (M1.1), hematopoietic precursors (M3.3 and M5.3), and neutrophils (M5.15). Conversely, they demonstrated significant under-expression of modules linked to T cells (M4.1, M6.15 and M6.19), cytotoxicity/NK cells (M3.6 and M4.15), B cells (M4.10), and lymphoid lineage (M4.7 and M6.9). Transcript composition of these modules is summarized online (http://www.biir.net/public_wikis/module_annotation/V2_Trial_8_Modules). These findings were confirmed in the test set as shown by significant correlation of module expression between training and test sets (Figure 1F, p<0.0001, Spearman R = 0.94) demonstrating the consistency of these observations.

### The blood signature of *S. aureus* infection demonstrates significant heterogeneity

As the clinical presentation of individual patients was diverse, once we defined the global whole blood response to *S. aureus* infection, we then aimed to characterize how the variation in anatomical site, dissemination of the infection and bacterial isolate (Table 2) affect the signatures. Because the training and test sets yielded similar modular fingerprints (Figure 1F), they were merged into one dataset for subsequent analysis.

**Table 2:** Infection localization and clinical presentation distribution for training and test sets.

		Definition	Training(n = 40)	Test(n = 59)	Total(n = 99)
Localization	Local	SST abscess or cellulitis	0 (0%)	10 (16.9%)	10 (10%)
	Invasive	Bacteremia, pneumonia, osteomyelitis, meningitis	33 (82.5%)	41 (69.5%)	74 (74.8%)
	Disseminated	Bacteremia+2 sites of infection	5 (12.5%)	8 (13.6%)	13 (13%)
	Unclassified		2 (5%)	0 (0%)	2 (2%)
ClinicalPresentation	SST Abscess	Skin abscess, no bacteremia	0 (0%)	10 (16.9%)	10 (10%)
	Osteoarticular	Bacteremia and osteomyelitis or suppurative arthritis	29 (72.5%)	27 (45.8%)	56 (56.6%)
	Pneumonia		6 (15%)	5 (8.5%)	11 (11.1%)
	Unclassified		5 (12.5%)	17 (28.8%)	23 (23.2%)

**Figure 2:**
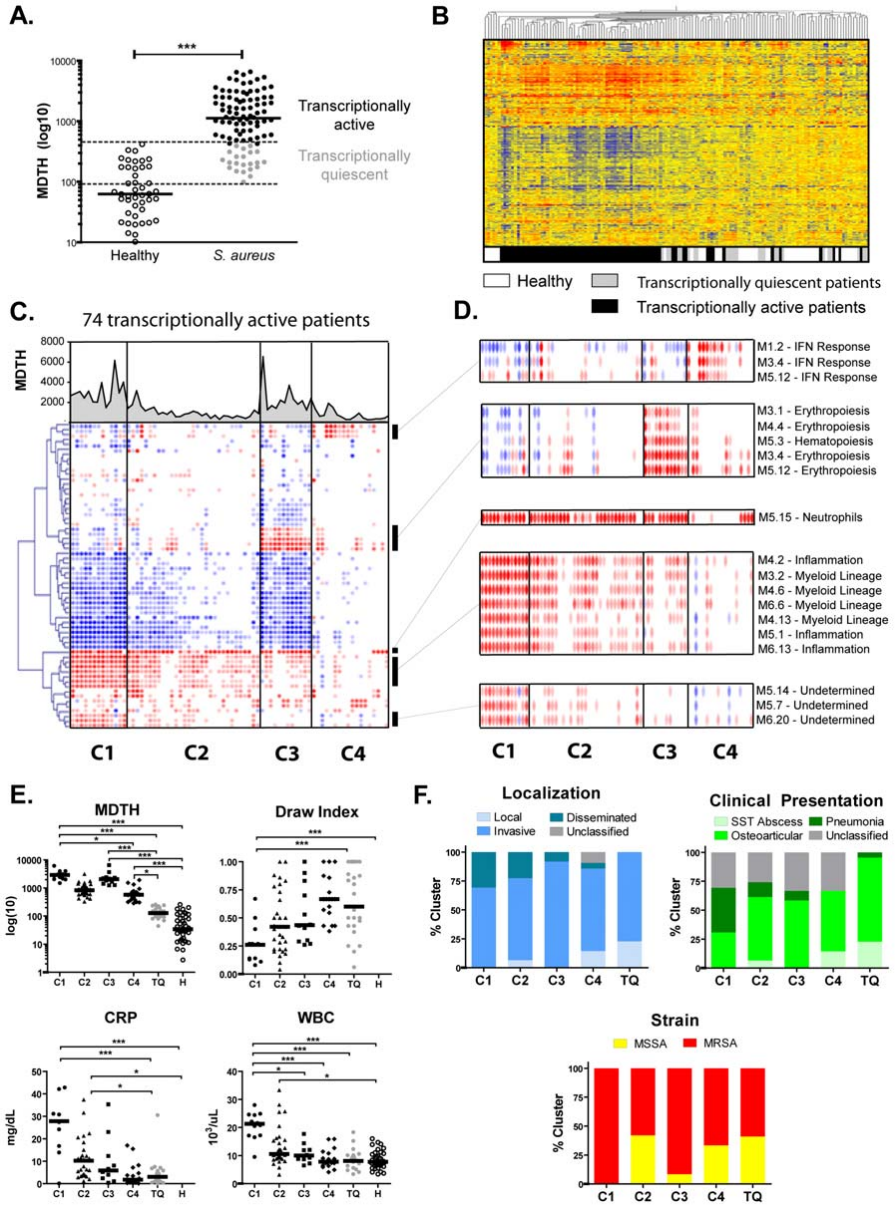
Individual analysis identifies heterogeneous components of the blood signature to *S. aureus*. **A.** Column scatter plot representing the distribution of individual molecular distance to health (MDTH) in healthy controls and *S. aureus* patients. The list of all transcripts composing the modules was used as reference to calculate individual MDTH (***: p<0.001, Mann-Whitney). Horizontal bars represent the group median. Patients with MDTH within healthy range (n = 25) were categorized as transcriptionally quiescent (TQ) and represented in grey. **B.** Unsupervised hierarchical clustering of the 10,972 transcripts expressed in at least one of the 143 samples (2-fold normalized, 100 difference in raw data) from the combined training and test sets. **C.** The modular signature was derived for individual transcriptionally active patients (n = 74) as compared to the median of the healthy control group for the corresponding patient set (training or test). Four major clusters (C1 through C4) of patients were obtained by K-means clustering and reorganized into a single heatmap, with modules in rows and patients in columns. Molecular distance to health for individual samples is represented as a line chart on top of the heatmap. **D.** Zoom on modules with specific over-expression patterns across the four clusters. **E.** MDTH and clinical lab measurements distribution by cluster. Five or six-group non-parametric ANOVA (Kruskal-Wallis) with Dunn's post-hoc test was applied. (*: p<0.05, **: p<0.01, ***: p<0.001). **F.** Bar charts representing the percent distribution of infection localization, clinical presentation and bacterial strain for the five clusters of patients identified.

Our initial step was to examine the heterogeneity in the signatures and then determine which factors could be associated with such variation. To this end, we first calculated the molecular distance to health, or MDTH for each individual patient. The MDTH is a score that measures the global transcriptional perturbation in each patient compared to the median of healthy controls. Thus by summarizing the overall transcriptional activity in one score, the MDTH facilitates the correlation with clinical parameters [12], [17]. Overall, as expected, the median MDTH of patients was significantly higher than that of healthy controls (Figure 2A, p<0.001). However, 25 patients had MDTH scores that were within the range of healthy controls MDTH (3–259). These patients were flagged as transcriptionally quiescent (TQ) and separated from transcriptionally active patients (n = 74). Unsupervised hierarchical clustering of the 10,972 transcripts expressed in at least one of the 143 subjects grouped these quiescent patients with healthy controls (Figure 2B). When analyzed from a clinical perspective the majority of these patients presented with mild and local infections with low C-reactive protein (CRP) and the samples were obtained late in the course of the infection as reflected by an elevated mean draw index (0.64) (see methods).

To assess the qualitative heterogeneity of transcriptionally active patients (n = 74; MDTH scores >259), individual modular fingerprints were derived and k-means clustering was used to further identify modular expression patterns. Four major transcriptional active patient clusters (C1 to C4) were identified (Figure 2C, Figure 2D) [18] (Figure S3). Group module expression was derived for each cluster (Figure S4). Cluster C1 included 13 patients with a high mean MDTH (3,003) and strong over-expression of myeloid (M3.2, M4.6, M4.13, M6.6) and inflammation (M4.2, M5.1, M6.13) modules. Cluster C2 comprised 31 patients with a signature qualitatively similar to C1, but quantitatively dimmer as supported by the lower mean MDTH (973). Cluster C3 regrouped 12 patients with high MDTH (2,395) and low inflammatory signature, but over-expression of modules linked to erythropoiesis (M2.3, M3.1, M4.4, M6.18) and hematopoiesis (M5.3). C1 and C3 both displayed significant down-regulation of transcripts linked to B and T cells (Figure S5). Gene level analysis (Figure S6A) combined with PANTHER [19] ontology ranking (http://www.pantherdb.org) supported this observation as heme biosynthesis (Figure S6B) and porphyrin metabolism (Figure S6C) were the most enriched pathways in C3 versus C1. The top 100 transcripts differentially expressed between C1 and C3 are displayed in Table S4 and Figure S6D. Finally, cluster C4 was comprised 18 patients with lower mean MDTH (699). Interestingly, 11 of them displayed an IFN signature, although only one patient had concomitant CMV infection detected by viral culture.

The median values for different clinical findings and laboratory parameters were calculated for each cluster (Figure 2E, Table S5). In addition, we assessed whether the differences observed at the modular level were related to the time of sample collection in relation to the length of stay (draw index). Cluster C1 displayed a lower draw index, indicating that blood samples were collected at an earlier stage of hospitalization. Patients from C1 required a longer duration of hospitalization than other patients. Additionally, patients from C1 had significantly higher CRP, WBC, neutrophil and monocyte counts. Thus, these routine laboratory markers of inflammation corroborated the over-expression of myeloid and inflammatory modules.

The C3 patients showed decreased hemoglobin, hematocrit, and MCHC (Figure S6F, Table S5), suggesting an anemic state that might trigger erythropoiesis as indicated by the signature. The C3 erythropoietic signature overlapped with transcripts from CD71+ early erythroid precursors [20] (Figure S6D).

Then, we analyzed how infection site, clinical presentation, and type of bacterial strain were distributed among clusters (Figure 2F, Table S6). All patients in cluster C1 had invasive or disseminated disease, and a higher proportion of patients with pneumonia. Clusters C4 and TQ (transcriptionally quiescent) included most patients with skin and soft tissue abscesses, confirming the association between low MDTH and mild presentation.

### Bacterial factors had limited influence on the host expression profiles

Finally, to determine whether heterogeneity in bacterial virulence factors was associated with clustering patterns, the isolates from 63 patients were characterized (Figure S1). The majority (87%) of isolates tested were PVL-positive. Both clusters C1 and C3 displayed a higher percentage of MRSA isolates (100% and 92%) than the overall mean (67%), suggesting that clones of CA-MRSA USA300 might induce a stronger host response than the currently circulating MSSA clones. No difference in other bacterial characteristics such as agr locus type or genetic relatedness was observed between the four clusters. (Table 3, Table S7).

**Table 3:** Distribution of bacterial isolate characteristics by infection localization, presentation and cluster.

			Infection Dissemination		Clinical Presentation		Cluster	
			Fisher's ExactTest		Fisher's ExactTest		Fisher's ExactTest	
			TableProbability (P)	Pr< = P	TableProbability (P)	Pr< = P	TableProbability (P)	Pr< = P
	# Patients		-	-	-	-	-	-
	# Isolates		-	-	-	-	-	-
**Antibiotic Resistance**	MRSA		0.07	**>0.05**	0.06	**>0.05**	0.0016	**>0.05**
	MSSA							
**Agr Type**	agr I		0.08	**>0.05**	0.11	**>0.05**	2.60E-03	**>0.05**
	agr II							
	agr III							
**Genetic Relatedness**	USA 200		0.01	**>0.05**	0.02	**>0.05**	2.20E-07	**>0.05**
	USA 300							
	USA 400							
	USA 500							
	USA 700							
	USA 1100							
**Toxin Production**	PVL	+	0.12	**>0.05**	0.16	**>0.05**	1.40E-02	**>0.05**
		−						
	TSST/SEA/SEB/SEC	+	0.15	**>0.05**	0.17	**>0.05**	0.028	**>0.05**
		−						
	SEH/SEG	+	0.09	**>0.05**	0.18	**>0.05**	0.0019	**>0.05**
		−						

TQ: Transcriptionally Quiescent.

### Elements of the molecular signature correlate with laboratory parameters

**Figure 3:**
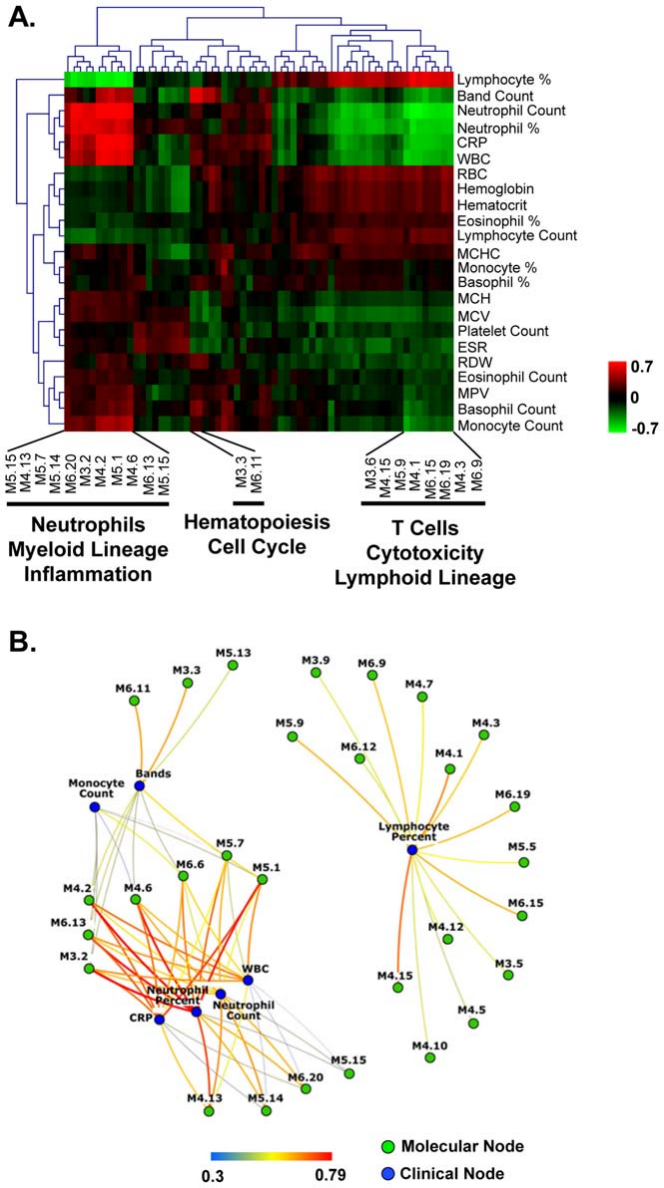
Specific module subsets correlate with laboratory results. **A.** Heatmap representing correlation (Spearman R) between module percent expression in columns and continuous laboratory parameters in rows. Hierarchical clustering (Euclidian distance) was applied in both dimensions. **B.** Connection network representing correlation between molecular nodes (modules) in blue and clinical nodes (laboratory parameters) in green. Spearman R correlation greater than 0.3 are represented.

We then asked whether patient's molecular profiles correlated with clinical laboratory parameters commonly used to assess clinical disease status (Table S8). The MDTH positively correlated with neutrophil counts, white blood cell counts, C-reactive protein, band neutrophil counts, red blood cell distribution width, and monocyte counts. MDTH inversely correlated with relative lymphocyte counts, red blood cell count, hemoglobin concentration, mean corpuscular hemoglobin (MCH) concentration, and hematocrit. Correlations were assessed on a module-by-module basis (Figure 3A). Inflammatory modules positively correlated with neutrophil counts, CRP and WBC while modules linked to adaptive immunity negatively correlated with these parameters. Hematopoiesis (M3.3) and cell cycle (M6.11) modules correlated with absolute band (immature neutrophils) count. Significant correlations between clinical nodes and molecular nodes are summarized as a network (Figure 3B).

### Time of blood sampling, infection dissemination, and clinical presentation influence the transcriptional signature

**Figure 4:**
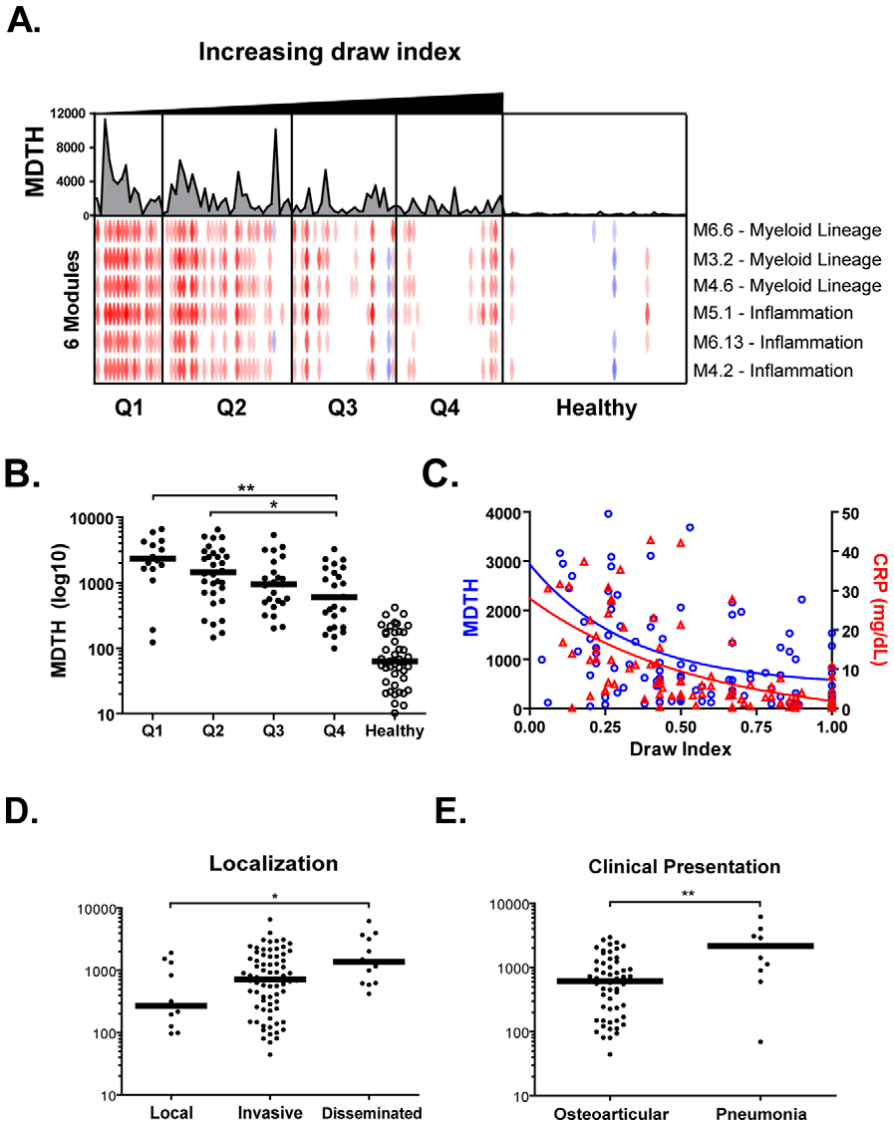
Patient signature varies with blood draw index, dissemination and clinical presentation. **A.** Patients were organized in four quarters Q1 through Q4 based on blood draw index (ratio draw day/hospitalization duration). A low draw index signifies proximity to hospital admission while a high draw index signifies proximity to discharge. Transcripts differently expressed between the four draw index quarters were selected by non-parametric ANOVA (Kruskal-Wallis, p<0.01, Benjamini-Hochberg false discovery rate) and represented as a heatmap (red, yellow, blue). The same statistical filter was applied at the module level (red, white, blue heatmap below). Individual MDTH was represented above as a line chart. **B.** Column scatter plot of individual MDTH per blood draw index quartile. Horizontal bars represent group median. Non-parametric ANOVA (Kruskal-Wallis) with Dunn's post-hoc test was applied. **C.** Non-linear regression model (one-phase decay) of MDTH (left Y-axis) and CRP (right Y-axis) as a function of blood draw index. **D.** Column scatter plot of individual MDTH per infection localization group. **E.** Column scatter plot of individual MDTH per clinical presentation group. Horizontal bars represent the median value for each group. Non-parametric ANOVA (Kruskal-Wallis) with Dunn's post-hoc test was applied between patient groups.

To determine whether the type and dissemination of the disease as well as time of sample collection influenced the genomic fingerprint, a supervised analysis was conducted to compare transcriptional signatures according to: 1) time in the course of the infection as defined by draw index quarters; 2) infection site; and 3) types of invasive clinical presentations. Three myeloid lineage and three inflammation related modules were differentially regulated from quarter to quarter (Figure 4A). The MDTH also displayed a decreasing trend (Figure 4B) that was paralleled by the decrease in CRP (Figure 4C). MDTH increased as the infection became more disseminated (Figure 4D), which was most evident during the first half of the hospitalization period (Figure S7A, Figure S7B), and was paralleled by increased CRP and hospitalization duration (Table S9).

Next, we analyzed patients according to the type of clinical presentation. Patients with pneumonia had a higher median MDTH than patients with osteoarticular infections as well higher WBC, neutrophil and monocyte counts (Figure 4E, Figure S8A, and Table S10).

### Patients with osteoarticular infections display transcripts linked to activated blood coagulation that are not present in patients with pneumonia

**Figure 5:**
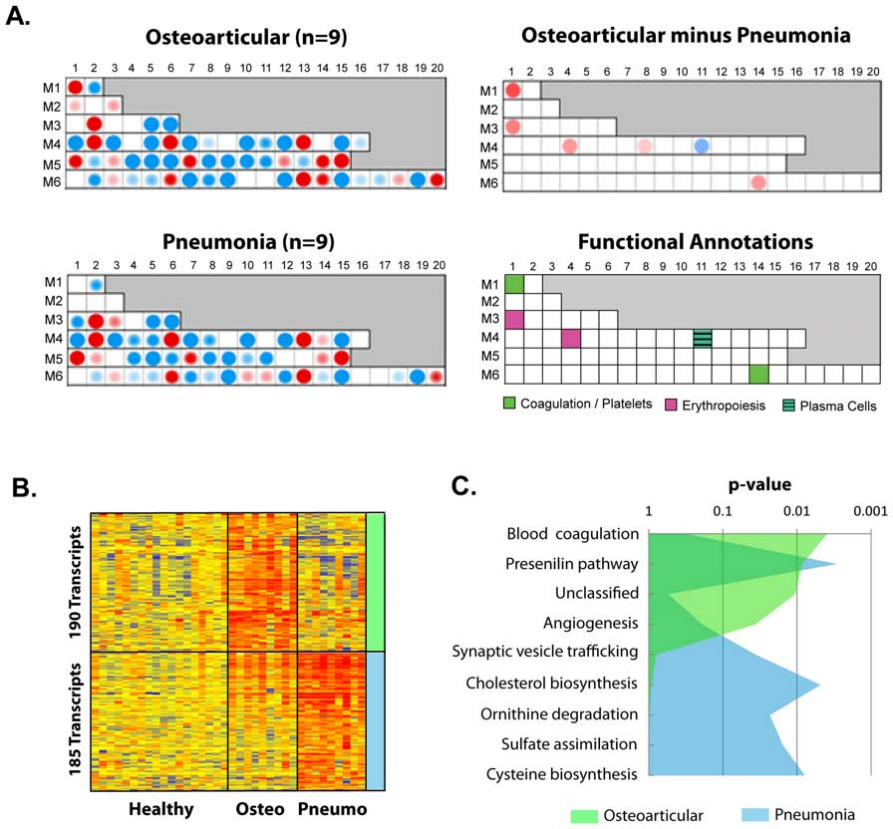
The osteoarticular infection signature displays increased blood coagulation. We compared the transcriptional signatures from patients with pneumonia and patients with osteoarticular infections. To properly balance osteoarticular and pneumonia groups, patients with pneumonia with a draw index less than 0.75 (nine patients) were selected (active disease). Nine patients with osteoarticular infection were selected with matching MDTH so that global quantitative signature was equivalent between the two groups. Nine healthy controls were selected from the training and nine from the test set (18 healthy controls in total) as reference. **A. Top left panel:** mean module map for the nine patients with osteoarticular infections compared to the 18 healthy controls. **Bottom left panel:** mean module map for the 9 patients with staphylococcal pneumonia compared to the 18 healthy controls. **Top right panel:** substraction map of osteoarticular infections minus pneumonia. Only differences greater than 40% are represented. **Bottom right panel**: Annotation legend for modules identified. **B.** Heatmap representing genes differentially expressed (t-test, <0.05, no correction) between osteoarticular infections and pneumonia (hierarchical clustering, Pearson). 190 genes were upregulated 1.5-fold or more in osteoarticular infections versus pneumonia and healthy controls. 185 genes were upregulated 1.5-fold or more in pneumonia versus osteoarticular infections and healthy controls. **C.** Area chart representing PANTHER comparison for pathway enrichment between the two lists from C.

To assess transcriptional differences between patients with distinct clinical presentations we selected nine patients with osteoarticular infection and compared them to nine patients with pneumonia matched for MDTH (Figure S8B). Eighteen healthy controls (nine from each training and test sets) were used as reference. Module fingerprints identified over-expression of the coagulation cascade (M1.1) and platelet adhesion (M6.14) in patients with osteoarticular infection but not pneumonia (Figure 5A). From the 385 genes differently expressed (Figure 5B), PANTHER analysis for pathway enrichment (Figure 5C) identified blood coagulation as the most significant pathway over-expressed in osteoarticular infection, and cholesterol biosynthesis over-expressed in pneumonia. No significant correlations were observed between bacterial isolates (Table S7) and clinical presentation.

## Discussion

This study is the first analysis of whole blood transcriptional profiles in pediatric patients with acute community-associated *S. aureus* infections. Both conserved and heterogeneous elements of the transcriptional signature were identified, which correlate with time elapsed since hospitalization, bacterial dissemination, and the type of clinical presentation. Supporting our earlier study on PBMC [21], the whole blood signature was characterized by significant over-expression of myeloid lineage and inflammation transcripts, and under-expression of lymphoid lineage transcripts. This may reflect the large expansion of circulating neutrophils and monocytes during the acute phase of infection.

While all patients share a global signature to *S. aureus* infection, analysis of fingerprints of individual patients revealed heterogeneous elements of the molecular signature. The major transcriptional patterns identified included a pro-inflammatory myeloid signature, linked to sampling early in the course of infection, high neutrophil and monocyte counts, and elevated CRP (C1). Some patients displayed an erythropoiesis fingerprint with limited myeloid components (C3), which likely represents an increased release of hematopoietic precursors from the bone marrow during acute infection [22]. This erythropoiesis signature was observed in systemic onset juvenile idiopathic arthritis (SOJIA) [20] and was proposed to reflect the expansion of immature precursor cells and ineffective erythropoiesis. The latter results in accumulation of iron in tissues [23], which supports bacterial survival. It was suggested that erythropoietin (EPO) inhibits NF-kB and TNFa-mediated pro-inflammatory pathways [24], which could explain why C3 patients displayed limited pro-inflammatory signature. This could represent a bacterial survival mechanism, whereby increased erythropoiesis would prevent the development of an adequate pro-inflammatory response and subsequent bacterial clearance. Despite increased numbers of neutrophils, we did not detect consistent changes in transcripts linked to the interferon response, as previously observed in patients with active pulmonary tuberculosis [12]. Fifteen out of 99 patients displayed over-expression of the three IFN modules but tested negative for concomitant viral infections. It may reflect IFN activation to counter arrest the bacteria-induced pro-inflammatory milieu [25] in the later stages of infection.

Currently, there are no established laboratory markers that either objectively define the extent of clinical disease severity in patients with *S. aureus* infections or monitor the spread of the infection to multiple organs [8]. Transcriptional profiling highlighted global quantitative differences between patients with local or disseminated disease, supporting the value of microarrays and the quantitative MDTH genomic score to monitor the spread of infection.

Additionally, the signatures provided unique transcriptional information on the pathogenesis of different clinical syndromes. Patients with osteoarticular infections displayed over-expression of transcripts linked to blood coagulation, a finding possibly related with the increased systemic coagulation and deep venous thrombosis observed in patients with musculoskeletal infections [26]. Additionally, patients with pneumonia displayed over-expression of genes involved in cholesterol synthesis, which might be involved in neutrophil recruitment to the lung [27]. Importantly, the location and extent of disease dissemination impacted transcriptional profiles to a greater extent than molecular characteristics of the *S. aureus* strains. This suggests that either: 1) the early innate response is directed towards features of *S. aureus* conserved across strains; 2) the human response is specifically tailored to the infection site; 3) staphylococcal gene expression differs based on the site of disease.

Despite the qualitative variability of the host response, the intensity of the signature decreases as patients get closer to discharge, regardless of the localization of the infection or the type of clinical presentation, suggesting that this assay has potential to monitor the clinical course of infection. Future studies should focus on the diagnostic and prognostic value of this approach in identifying patients at risk for infection dissemination and eventually determine its value in guiding therapeutic decisions.

## Materials and Methods

### Ethics Statement

This study was conducted according to the principles expressed in the Declaration of Helsinki. The study was approved by the Institutional Review Boards of the University of Texas Southwestern Medical Center and Children's Medical Center of Dallas (IRB #0802-447) and Baylor Institute of Immunology Research (BIIR, IRB # 002-141). Informed written consent was obtained from legal guardians and informed assent was obtained from patients 10 years of age and older prior to any study-related procedure.

### Patient characteristics

Blood samples from 99 patients hospitalized with community-acquired *S. aureus* infection and 44 healthy controls were collected in tempus tubes (Applied Biosystems, PN 4342792). Patients represented the clinical spectrum of acute *S. aureus* infection, including skin and soft tissue infection, bacteremia, osteomyelitis, suppurative arthritis, pyomyositis, pneumonia with empyema, and disseminated disease defined as bacteremia and the involvement of 2 different anatomical sites. Patients with a diagnosis of toxic shock syndrome, polymicrobial infections, or treated with corticosteroids in the preceding four weeks were excluded. Viral direct fluorescent antibody testing and/or culture of the nasopharynx was performed in all patients and healthy controls to exclude concomitant viral infections. Patient demographic data and clinical characteristics are summarized in Table S1 and Table S2. The median duration of hospitalization was ten days (range: 1–98 days). The median time from patient hospitalization to blood sample acquisition was five days (range: 1–35 days).

### Patient classification

The study cohort of 99 patients and 44 healthy controls was divided into independent training (40 patients, 22 healthy controls) and test sets (59 patients, 22 healthy controls) (Table 1). Patients were categorized according to three schemes (Table 2) based on assessment by an independent clinician who was blinded to the transcriptional data: i) by localization of infection, defined as local (n = 10), invasive (n = 74) or disseminated (n = 13); ii) by clinical presentation, separating patients with skin and soft tissue infection with negative blood culture (n = 10), patients with osteoarticular infections (n = 56), and patients with pneumonia (n = 11).

### Sampling time: Draw index and hospitalization quarter

This cross-sectional study included samples drawn at different days during hospitalization. To assess the influence of the time the sample was obtained during the course of the infection we calculated a draw index, a numeric score between 0 and 1 calculated as the ratio of the blood draw day over the duration of hospitalization. Accordingly, samples were classified according to the hospitalization quarter (0≤Quarter 1<0.25≤Quarter 2<0.50≤Quarter 3<0.75≤Quarter 4).

### Characterization of Bacterial Isolates

Bacterial isolates from 63 patients were recovered from blood culture, synovial fluid, or abscesses. Single colonies were selected and sub-cultured. *S. aureus* was confirmed by nuclease PCR and isolates were tested for methicillin resistance by mecA PCR. SCCmec typing of MRSA isolates was performed by classifying the ccr and mec complexes [28]. agr locus typing was performed and genetic relatedness was determined by repetitive-element, sequence-based PCR (rep-PCR). Gene encoding of toxins was detected by traditional PCR [28], [29] (Figure S1, Table S3).

### RNA Preparation and Microarray Hybridization

RNA was processed as described elsewhere [12]. The data are deposited in the NCBI Gene Expression Omnibus (GEO, http://www.ncbi.nlm.nih.gov/geo, GEO Series accession number GSE30119).

### Batch Correction

To prevent batch effect between training and test sets, principal variance component analysis (PVCA) was conducted using JMP Genomics (SAS Institute, Cary, NC) to identify sources of batch effect. Cohort number accounted for 58% of the variability observed (Figure S2A) and scatter plot visualization (Figure S2B) with ellipsoids (Figure S2C) revealed strong segregation of samples based on cohort. The batch correction algorithm CombatR [30] was used on the cohort variable to reduce its contribution to the global variance. The batch effect from the cohort was reduced to approximately 0% (Figure S2D, Figure S2E and Figure S2F).

### Module Framework Development

This analysis strategy has been described elsewhere [16]. A set of 62 transcriptional modules derived from 410 whole blood gene expression profiles was applied to the dataset described herein. Modules were annotated with Ingenuity Pathway Analysis (IPA) (Ingenuity Systems, Redwood City, CA), Pubmed, iHOP, and Novartis Gene Atlas (http://biogps.gnf.org) databases. Module transcript content and annotations are available online (http://www.biir.net/public_wikis/module_annotation/V2_Trial_8_Modules).

### Module-level Analysis

Gene expression levels were compared between patients and healthy controls on a module-by-module basis. The percentage of transcripts showing significant differences (Mann-Whitney, p<0•05) in expression was used as an indicator of module activity. Modules containing transcripts with increased expression were represented on a red scale while those containing transcripts with decreased expression were represented on a blue scale. Modules with 15% or less transcripts with significant change in abundance compared to healthy controls were not displayed.

## Supporting Information

Figure S1 **Characterization of the 63 clinical bacterial isolates.** **A.** Pie charts representing distribution of methicillin resistance, agr locus type and genetic relatedness for the 63 bacterial isolates isolated from culture. **B.** Bar chart representing the percentage of isolates positive for the measured toxins.
(TIF)

Figure S2 **Batch correction for the two cohorts of patients used as training and test sets.** **A.** Pie chart of percent contribution of individual parameter to variance from principal variance component analysis (PVCA) before CombatR correction. **B.** Scatter plot representing the segregation of sample by cohort before CombatR correction. **C.** Elliptical fit of scatter plot data from B. **D.** Pie chart of individual parameter's percent contribution to variance from PVCA after CombatR correction for cohort. **E.** Scatter plot representing the segregation of sample by cohort after CombatR correction. **F.** Elliptical fit of scatter plot data from E.
(TIF)

Figure S3 **Determination of the best number of K-means clusters from individual patient module expression.** An appropriate K was chosen for clustering this dataset using an information theoretic approach called the “jump method”. First, the data was clustered for all K 1 to 5, inclusive. **A.** The distortion of each clustering was calculated. In this instance, we chose to approximate the covariance matrix with the identity matrix so the distortion is simply the mean squared error. **B.** Next, the transformed distortion for each K is calculated by raising the distortion to a power of -(# of dimensions/2). **C.** Finally, the Jump at each K is calculated as follows: Jump_K = TransformedDistortion_K - TransformedDistortion_K-1. The maximum Jump is used to select K, in this case, K = 4.
(TIF)

Figure S4 **Module signature per cluster of patients.** Module signature was derived for each cluster of molecularly active patients (C1 to C4) and the group of transcriptionally quiescent (TQ) patients as compared to the combined 44 healthy controls.
(TIF)

Figure S5 **Cluster of under-expressed modules in the 74 molecularly active patients with** ***S. aureus*** **infection.**
(TIF)

Figure S6 **Cluster C3 displays an erythropoiesis signature.** **A.** Heatmap representing the 680 transcripts differentially expressed between clusters C1 and C3. Statistical comparison between C1 and C3 yielded 680 differentially expressed transcripts (non-parametric test, α = 0•01, Benjamini-Hochberg multiple testing correction, 2× fold change). Of these, 246 transcripts were over-expressed in C1 and 434 transcripts were over-expressed in C3. **B.** Area chart representing pathways significantly enriched (p<0•05) in the two gene lists identified in A according to PANTHER (http://www.pantherdb.org). **C.** Area chart representing biological processes significantly enriched (p<0•01) in the two gene lists identified in A according to PANTHER. **D.** Heatmap representing the top 100 transcripts over or under-expressed in C3 versus C1. **E.** Heatmap representing the expression of 87 transcripts specifically expressed in CD71+ erythroid precursors in the four patient clusters. **F.** Scatter plot of various CBC measurements for patients organized by transcriptionally active clusters C1 through C4 and transcriptionally quiescent (TQ) patients. The green rectangle represents the range of values for healthy children (two months to 18 years) recorded at Childrens Hospital, Dallas.
(TIF)

Figure S7 ***S. aureus*** **patient molecular distance to health varies with infection localization, clinical presentation and blood draw index.** A. MDTH as a function of blood draw index for patients grouped by disease localization. B. MDTH as a function of blood draw index for patients grouped by disease presentation. A non-linear regression statistical model (one-phase decay) was applied.
(TIF)

Figure S8 **Selection of osteoarticular infection and pneumonia patients for comparison.** **A.** Dot plot of neutrophil counts for patients grouped by clinical presentation. Horizontal bars represent the median value for each group. Non-parametric ANOVA (Kruskal-Wallis) with Dunn's post-hoc test was applied. **B.** Column scatter plot of individual MDTH for patients selected for comparison. Horizontal bars represent the median value for each group. Non-parametric testing (Mann-Whitney) was applied between the two groups of patients.
(TIF)

Table S1 **Training set subjects characteristics.**
(XLS)

Table S2 **Test set subjects characteristics.**
(XLS)

Table S3 **Characterization of bacterial isolates from 63 patients.**
(XLS)

Table S4 **Top 50 over-expressed genes in C1 vs. C3 (left) and C3 vs. C1 (right).**
(XLS)

Table S5 **Clinical parameters for patients grouped by transcriptional clustering.**
(XLS)

Table S6 **Disease localization, clinical presentation and strain distribution by cluster.**
(XLS)

Table S7 **Distribution of bacterial isolates characteristics by infection localization, clinical presentation and cluster.**
(XLS)

Table S8 **Spearman correlations between molecular distance to health (MDTH) and clinical laboratory parameters.**
(XLS)

Table S9 **Clinical parameters for patients grouped by infection localization.**
(XLS)

Table S10 **Clinical parameters for patients grouped by clinical presentation.**
(XLS)
