# Supplementary figures and images for: Host Immune Transcriptional Profiles Reflect the Variability in Clinical Disease Manifestations in Patients with Staphylococcus aureus Infections
Source: PLoS One. 2012 Apr 4;7(4):e34390. doi: 10.1371/journal.pone.0034390 (PMC3319567; doi:10.1371/journal.pone.0034390)

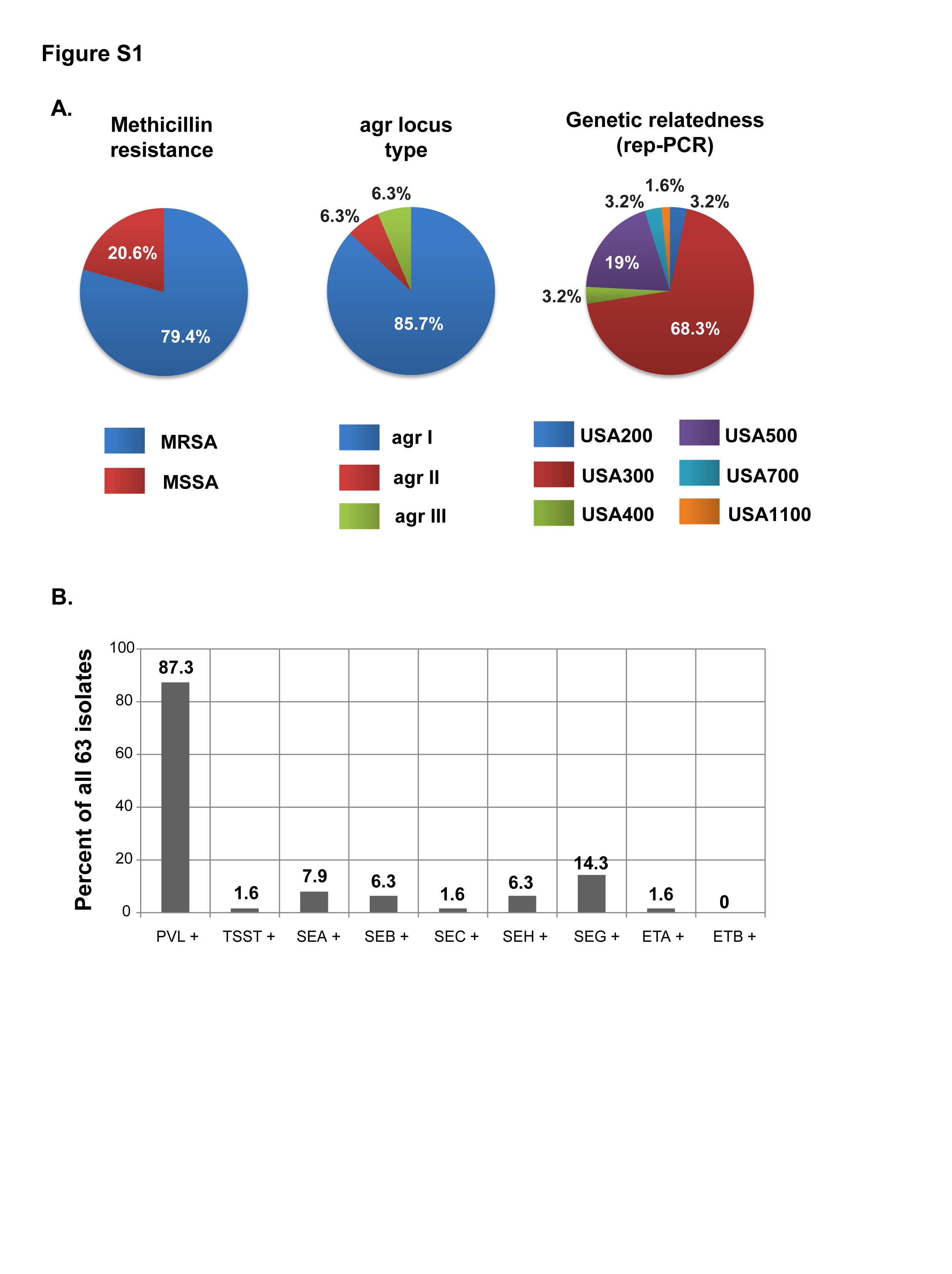

Supplement: Figure S1 — Characterization of the 63 clinical bacterial isolates. A. Pie charts representing distribution of methicillin resistance, agr locus type and genetic relatedness for the 63 bacterial isolates isolated from culture. B. Bar chart representing the percentage of isolates positive for the measured toxins. (TIF) [file pone.0034390.s001.tif]

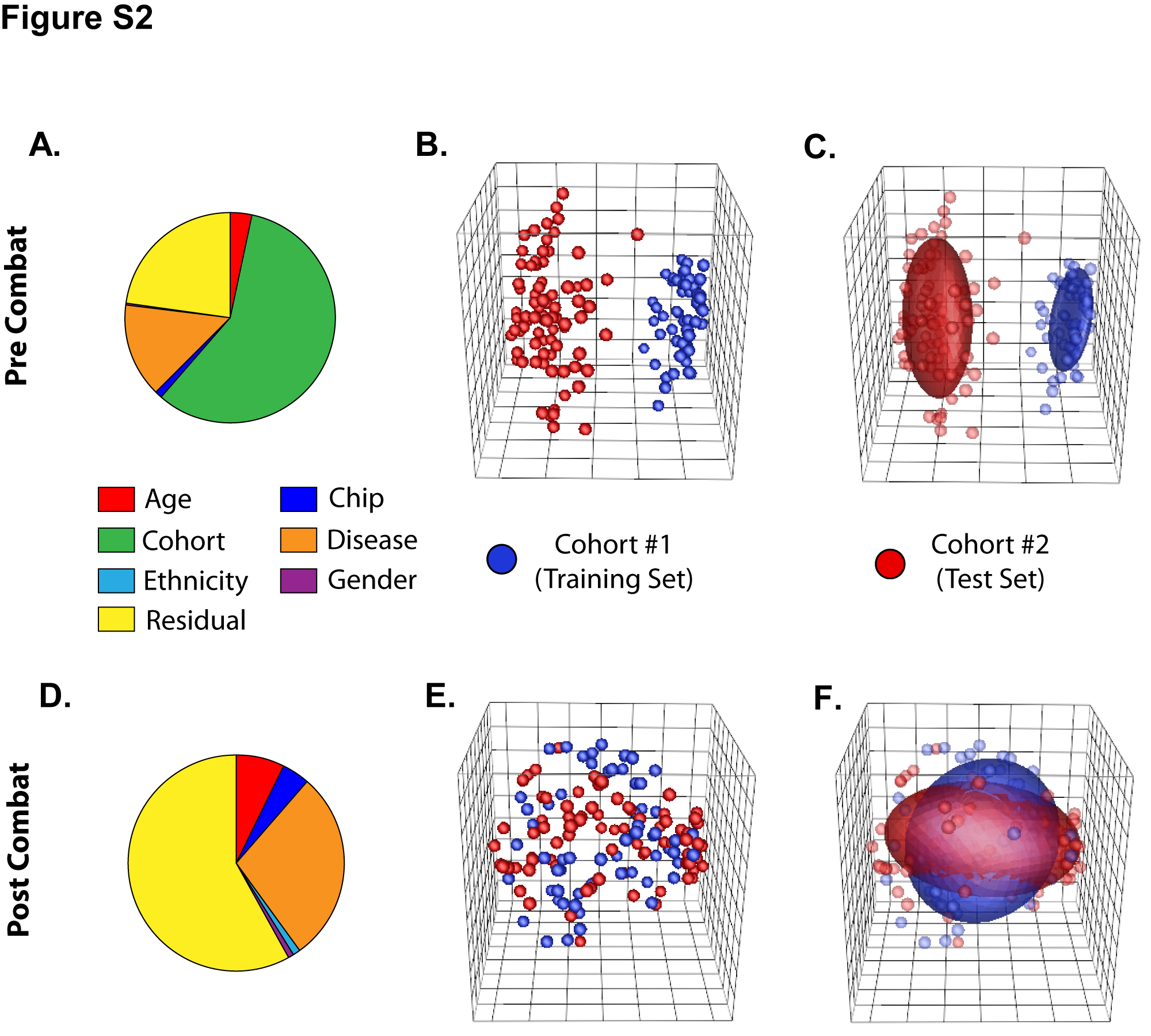

Supplement: Figure S2 — Batch correction for the two cohorts of patients used as training and test sets. A. Pie chart of percent contribution of individual parameter to variance from principal variance component analysis (PVCA) before CombatR correction. B. Scatter plot representing the segregation of sample by cohort before CombatR correction. C. Elliptical fit of scatter plot data from B. D. Pie chart of individual parameter's percent contribution to variance from PVCA after CombatR correction for cohort. E. Scatter plot representing the segregation of sample by cohort after CombatR correction. F. Elliptical fit of scatter plot data from E. (TIF) [file pone.0034390.s002.tif]

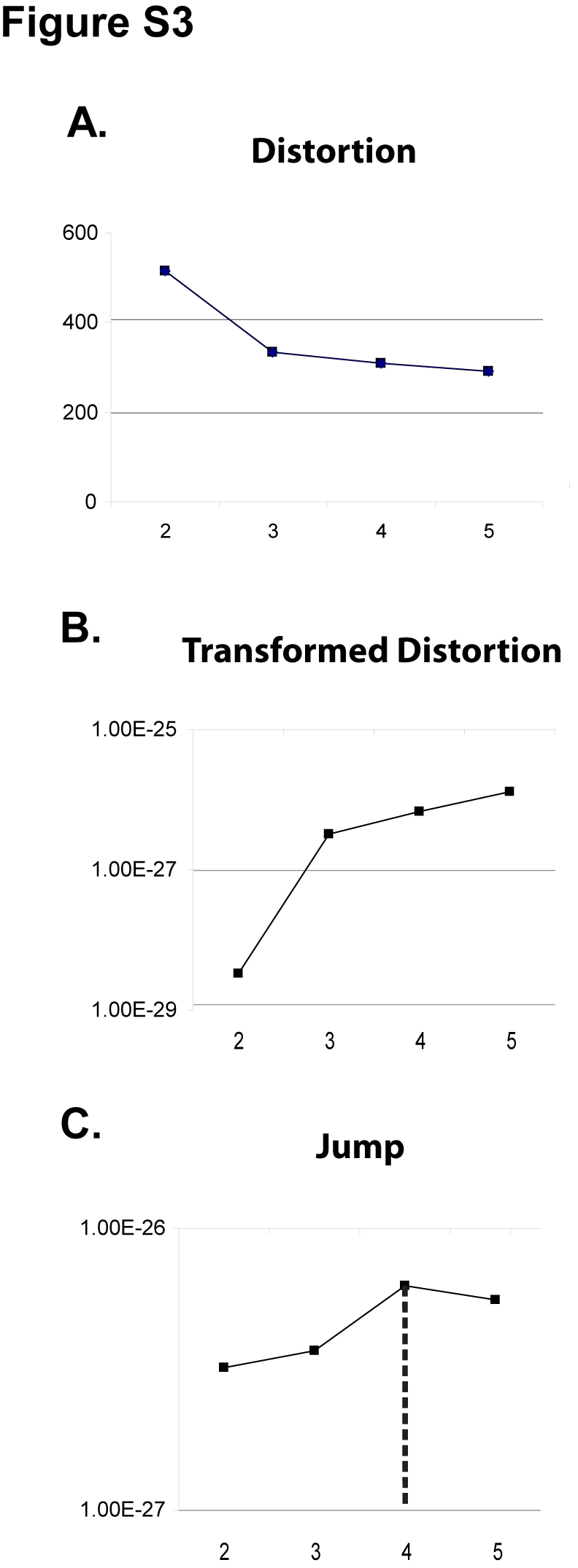

Supplement: Figure S3 — Determination of the best number of K-means clusters from individual patient module expression. An appropriate K was chosen for clustering this dataset using an information theoretic approach called the “jump method”. First, the data was clustered for all K 1 to 5, inclusive. A. The distortion of each clustering was calculated. In this instance, we chose to approximate the covariance matrix with the identity matrix so the distortion is simply the mean squared error. B. Next, the transformed distortion for each K is calculated by raising the distortion to a power of -(# of dimensions/2). C. Finally, the Jump at each K is calculated as follows: Jump_K = TransformedDistortion_K - TransformedDistortion_K-1. The maximum Jump is used to select K, in this case, K = 4. (TIF) [file pone.0034390.s003.tif]

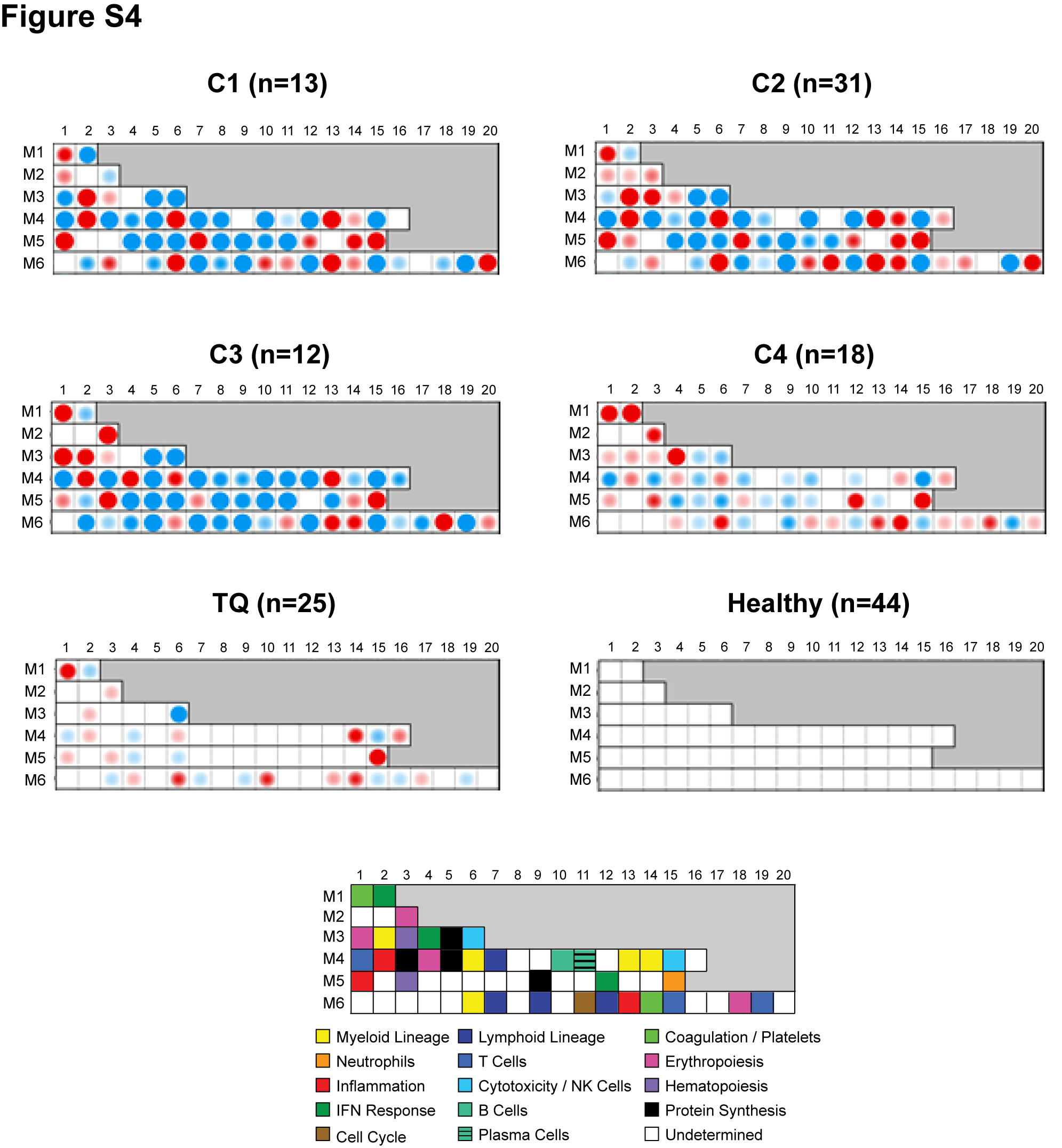

Supplement: Figure S4 — Module signature per cluster of patients. Module signature was derived for each cluster of molecularly active patients (C1 to C4) and the group of transcriptionally quiescent (TQ) patients as compared to the combined 44 healthy controls. (TIF) [file pone.0034390.s004.tif]

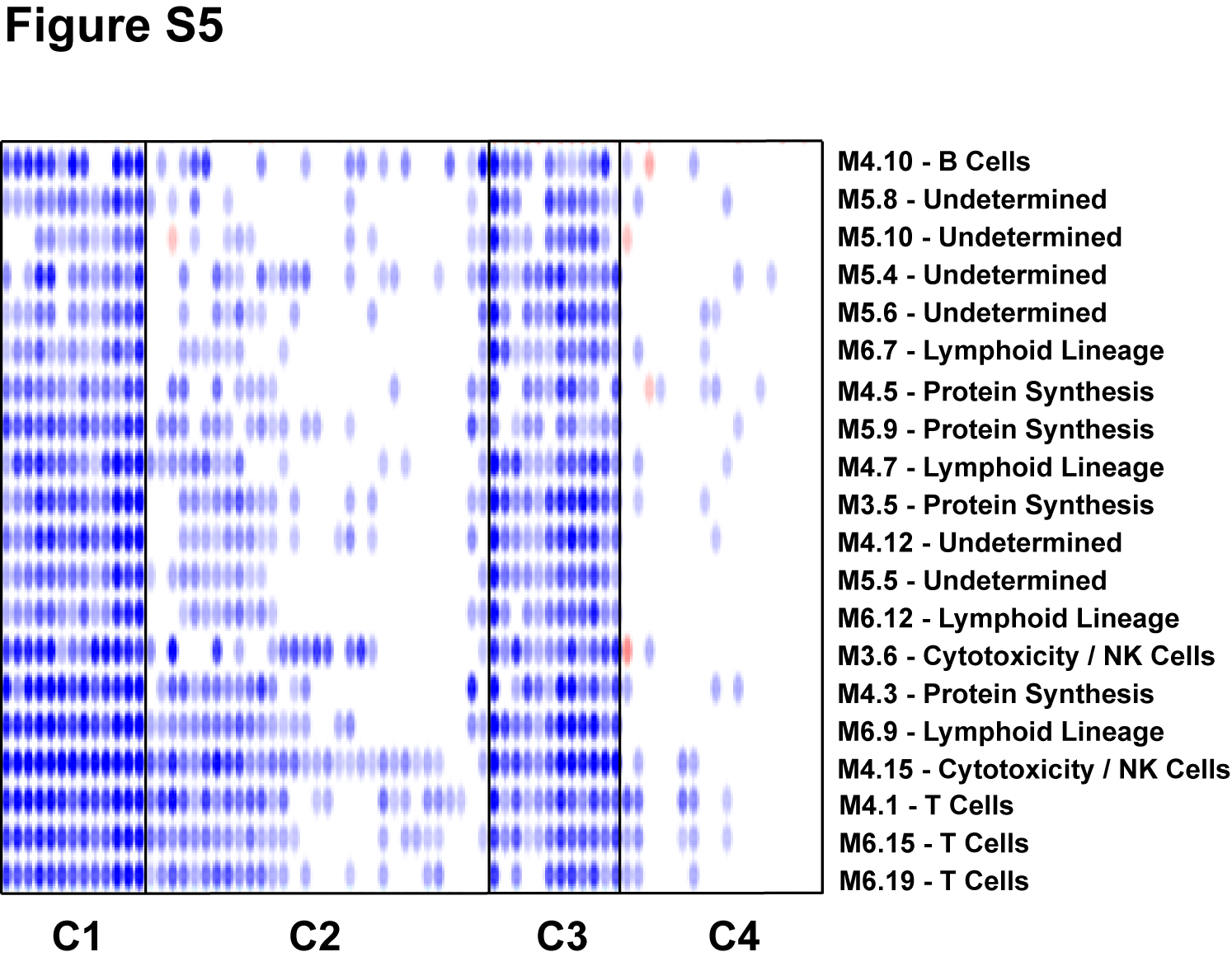

Supplement: Figure S5 — Cluster of under-expressed modules in the 74 molecularly active patients with S. aureus infection. (TIF) [file pone.0034390.s005.tif]

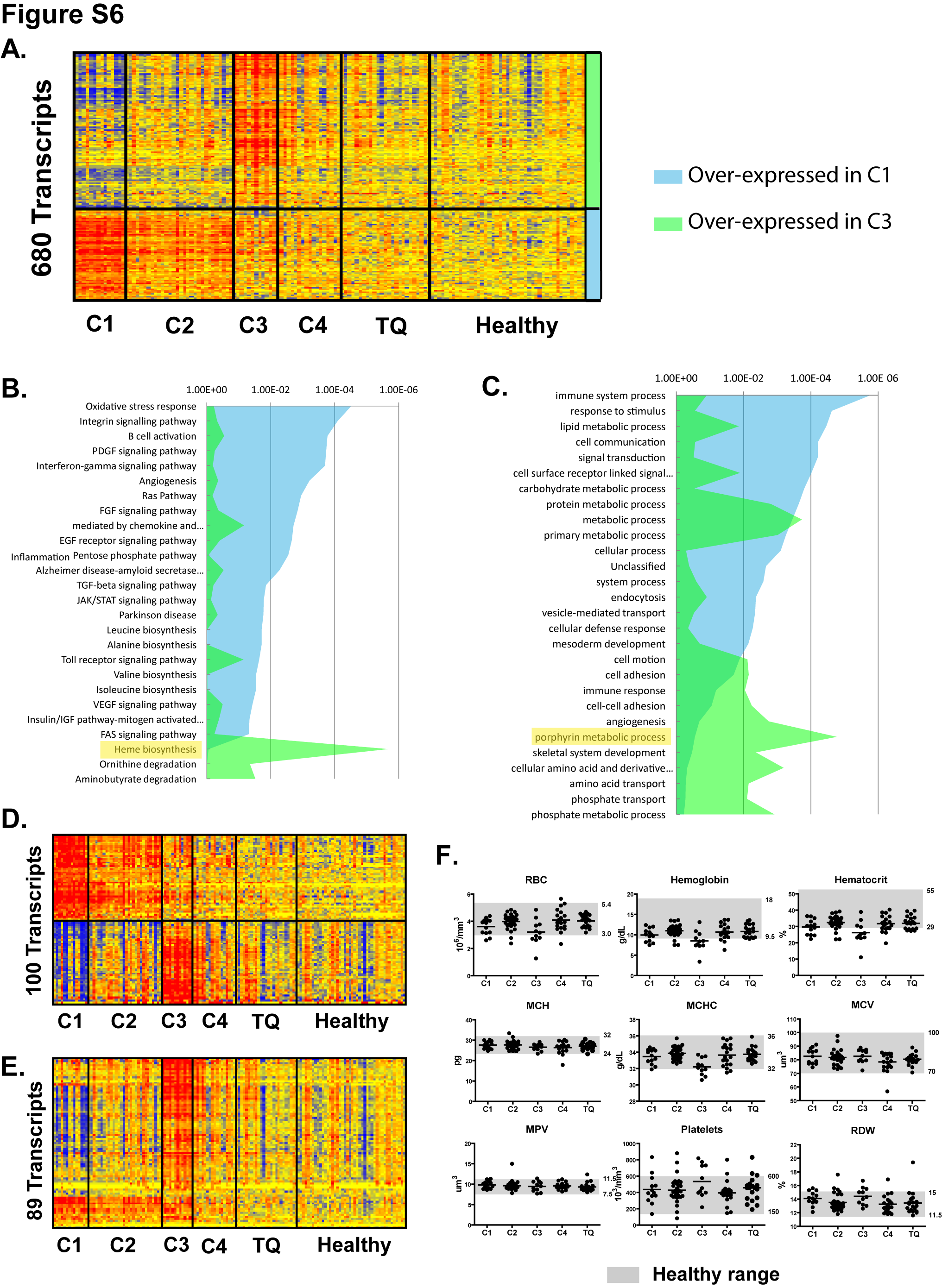

Supplement: Figure S6 — Cluster C3 displays an erythropoiesis signature. A. Heatmap representing the 680 transcripts differentially expressed between clusters C1 and C3. Statistical comparison between C1 and C3 yielded 680 differentially expressed transcripts (non-parametric test, α = 0•01, Benjamini-Hochberg multiple testing correction, 2× fold change). Of these, 246 transcripts were over-expressed in C1 and 434 transcripts were over-expressed in C3. B. Area chart representing pathways significantly enriched (p<0•05) in the two gene lists identified in A according to PANTHER (http://www.pantherdb.org). C. Area chart representing biological processes significantly enriched (p<0•01) in the two gene lists identified in A according to PANTHER. D. Heatmap representing the top 100 transcripts over or under-expressed in C3 versus C1. E. Heatmap representing the expression of 87 transcripts specifically expressed in CD71+ erythroid precursors in the four patient clusters. F. Scatter plot of various CBC measurements for patients organized by transcriptionally active clusters C1 through C4 and transcriptionally quiescent (TQ) patients. The green rectangle represents the range of values for healthy children (two months to 18 years) recorded at Childrens Hospital, Dallas. (TIF) [file pone.0034390.s006.tif]

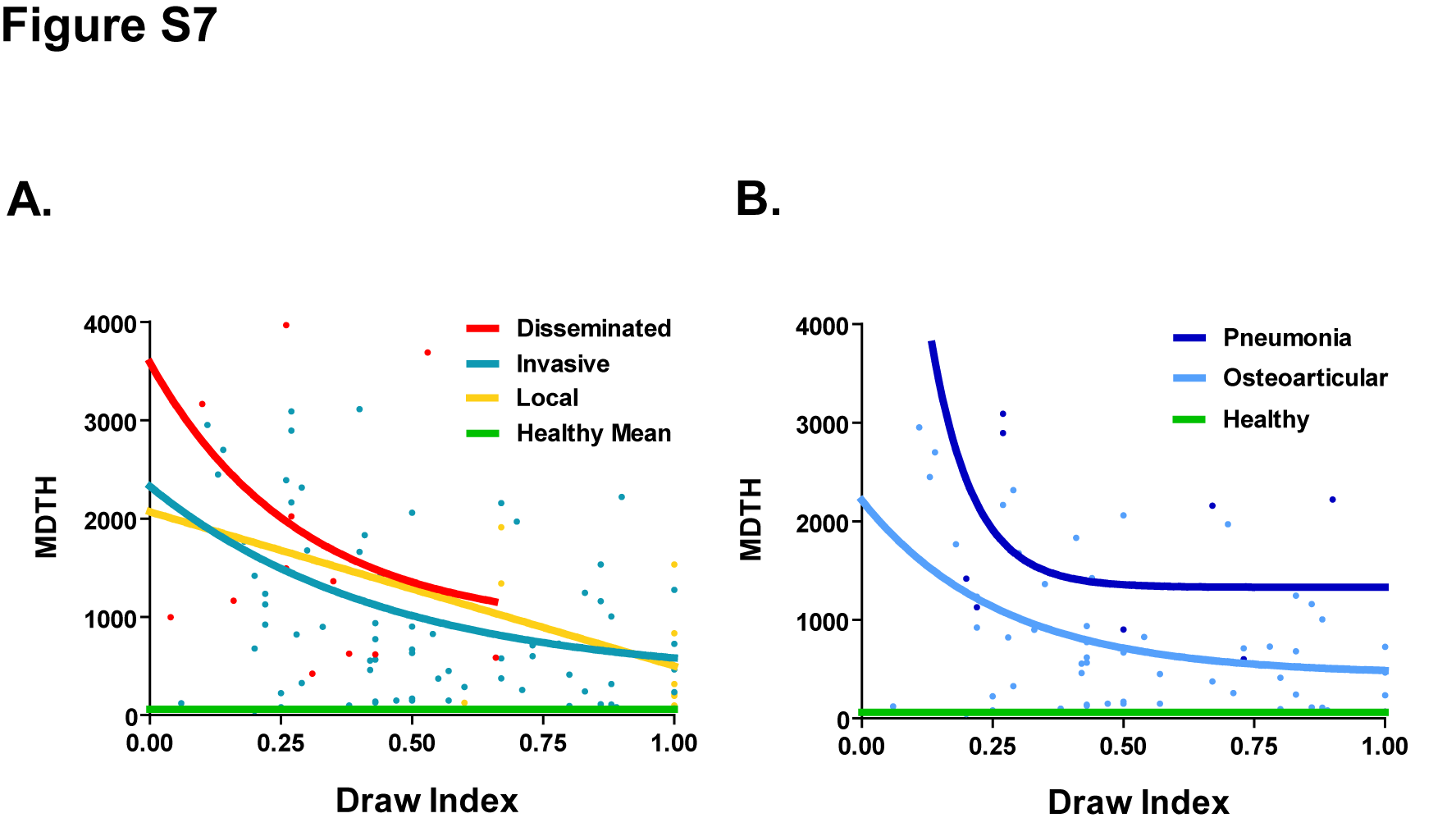

Supplement: Figure S7 — S. aureus patient molecular distance to health varies with infection localization, clinical presentation and blood draw index. A. MDTH as a function of blood draw index for patients grouped by disease localization. B. MDTH as a function of blood draw index for patients grouped by disease presentation. A non-linear regression statistical model (one-phase decay) was applied. (TIF) [file pone.0034390.s007.tif]

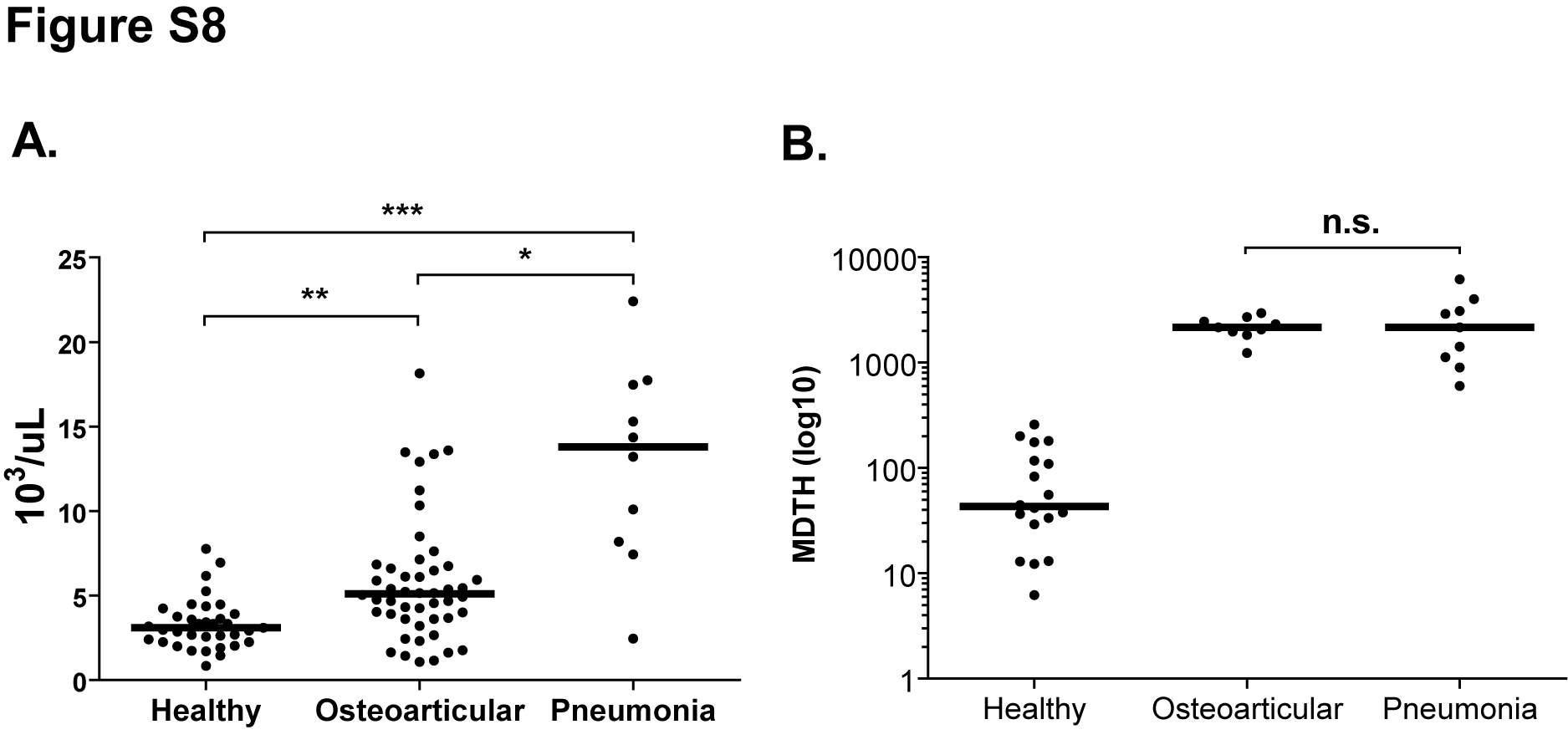

Supplement: Figure S8 — Selection of osteoarticular infection and pneumonia patients for comparison. A. Dot plot of neutrophil counts for patients grouped by clinical presentation. Horizontal bars represent the median value for each group. Non-parametric ANOVA (Kruskal-Wallis) with Dunn's post-hoc test was applied. B. Column scatter plot of individual MDTH for patients selected for comparison. Horizontal bars represent the median value for each group. Non-parametric testing (Mann-Whitney) was applied between the two groups of patients. (TIF) [file pone.0034390.s008.tif]
